# Reflections on training and management of junior radiologists through analysis of correction rates in emergency radiology reports

**DOI:** 10.1186/s12909-025-07573-1

**Published:** 2025-07-01

**Authors:** Hong Wu, Ming Li, Jingwen Chen, Xuhui Fan, Meijuan He, Zaixiong Ji, Han Wang, Xiaorui Yin

**Affiliations:** 1 Department of Radiology, Shanghai General Hospital, Shanghai Jiao Tong University School of Medicine, Haining Road 100, Shanghai, 200080 China; 2 R&D Center of Medical Artificial Intelligence and Medical Engineering, Shanghai General Hospital, Haining Rd. 100, Shanghai, 200080 China; 3 School of Computer Engineering, Jiangsu Ocean University, Lianyungang, 222005 Jiangsu China

**Keywords:** Radiology, Diagnostic errors, Emergency medical services, Medical education, Shift work schedule

## Abstract

**Purpose:**

Numerous studies have examined factors affecting junior radiologists’ interpretation accuracy, mostly focusing on Western countries. This study investigates the accuracy of radiology reports during emergency shifts in a Chinese hospital, considering shift times, examination types, radiologist experience, and patient factors.

**Materials and methods:**

From January 2023 to March 2024, we randomly selected emergency shifts and analyzed radiology reports initially interpreted by junior radiologists and reviewed by senior radiologists. This study compared the corrected rates between day (8 AM to 3:59 PM) and night shifts (8 PM to 7:59 AM), as well as between the first and second halves of each shift. Additionally, we examined the relationships between examination type and quantity, radiologist experience, patient age, and correction rates.

**Results:**

In 19 day shifts and 18 night shifts, 14 junior radiologists initially interpreted 8,338 reports. The corrected rate for daytime emergency reports (25.09%) was higher than for nighttime reports (21.91%, *p* = 0.236). Corrected rate in the second half of shifts was lower than in the first half, regardless of day (28.89% vs. 22.15%, *p* < 0.001) or night (22.89% vs. 19.05%, *p* = 0.008). Radiologist experience is significantly associated with corrected rates (*p* < 0.001), with elderly patients (29.85%) showing a higher rate of corrections compared to children (22.03%) and middle-aged patients (18.85%, *p* < 0.001).

**Conclusion:**

Correction rates of radiology reports vary under different training, different working hours, and different working models. Customizing management practices and training programs based on research findings is essential to improve accuracy and develop specific guidelines.

**Supplementary Information:**

The online version contains supplementary material available at 10.1186/s12909-025-07573-1.

## Introduction

Emergency radiology practice offers invaluable practical experience to junior physicians, providing them with extensive opportunities to develop and refine their diagnostic skills in a fast-paced, real-world setting. Emergency radiology (ER) requires radiologists to provide round-the-clock coverage, necessitating the accurate interpretation of imaging studies despite long working hours and high workloads. This presents a significant diagnostic challenge [1]. Numerous studies have identified factors affecting diagnostic accuracy, including time-related issues such as fatigue during night shifts or later stages of the same shift. These factors can reduce the accuracy of radiology reports and lead to adverse clinical events [2, 3]. Recent literature has shown that lower workloads are associated with reduced error rates [4]. Additionally, longer interpretation times, often linked to more complex diseases, correlate with higher error rates [5]. There is also evidence suggesting a positive association between the error rate and the radiologist’s age [6].

Most previous studies have focused on radiologist training and scheduling systems in Western countries, such as the United States, with varying results. This necessitates an understanding of how different training and scheduling systems impact the performance of radiologists in diverse medical environments. In China, radiologist training is standardized nationwide through the Standardized Residency Training (SRT) system, mandated by the Ministry of Health. This structured program shapes the diagnostic capabilities of junior radiologists and differs significantly from Western models [7]. First, the SRT program is a compulsory 3-year postgraduate curriculum designed to equip trainees with core theoretical knowledge and clinical skills in radiology, enabling them to independently perform diagnostic radiology, interventional therapy, and related research in a standardized manner. The training encompasses diagnostics for various imaging modalities—including X-ray, CT, MRI, ultrasound, and nuclear medicine—and interventional procedures. Trainees rotate through multiple clinical departments under senior supervision, focusing on mastering imaging diagnostics for common diseases across all organ systems.

Upon completing SRT, trainees join hospital staff as residents, continuing to undertake initial reporting for outpatient, emergency, and inpatient imaging cases. Each report is signed by the “initial interpreting physician” and must undergo secondary review and countersignature by a senior radiologist (“reviewing physician”), who revises and approves the content. The supervisory process ensures report accuracy and safeguards clinical safety. For junior radiologists, systematically comparing their initial reports with revised versions and incorporating feedback from senior radiologists provides an effective means of skill development.

The physician hierarchy in China is structured as follows: residents undergoing SRT (trainees), post-SRT residents (residents), attending physicians, associate chief physicians, and chief physicians. After completing the 3-year SRT, residents must work for at least 2 additional years before qualifying for the attending physician exam. In most Chinese hospital radiology departments, both residents and attending physicians are categorized as junior radiologists, while associate chief and chief physicians typically constitute senior radiologists. Advancement from attending to associate chief or chief physician generally requires at least 5 years of experience per stage, with promotion contingent on comprehensive evaluations of practical competence, research output, and clinical performance. As such, even radiologists with over 10 years of experience may not necessarily attain senior status. This contrasts sharply with Western systems. For example, U.S. radiologists complete a 4-year residency followed by 1–3 years of fellowship training for subspecialty certification, with the “attending physician” role being more analogous to China’s associate chief and chief physicians [2]. The UK system mandates 2 years of foundational training, 4–6 years of specialist training, and 1–2 years of subspecialty training before certification as a Consultant [8, 9]. In China, a narrow scope of practice confined to a single subspecialty is uncommon, and formal additional subspecialty (fellowship) training or certification is neither required nor actively pursued. China’s national SRT uniformity ensures that our single-center findings reflect broader practices in the country’s healthcare landscape, distinguishing the training and career progression pathways from those in Western nations.

To optimize management and training systems tailored to China’s healthcare system, local research is essential and can provide valuable insights for other countries facing similar challenges. Given the lack of comprehensive guidelines from radiology societies in both the United States and China on managing workflow to reduce error rates [4], as well as the inconsistencies in previous findings, this study investigates diagnostic discrepancies in ER reports among Chinese junior radiologists. The analysis considers various factors, including shift timing, types and volumes of examinations, and characteristics of radiologists and patients. The purpose is to provide critical insights for improving radiology practice and emergency patient care.

## Methods

Shanghai General Hospital Institutional Review Board approved this retrospective study (Approval No. 2024338) and waived the requirement for written informed consent. All patient data were anonymized and de-identified before analysis, with personal identifiers (e.g., names, medical record numbers) removed to ensure privacy. Data were stored on secure, password-protected servers in compliance with institutional data protection policies.

### Studies and facility

Our institution is a tertiary-level general medical center located in a satellite city of Shanghai. It features 980 inpatient beds and 48 clinical departments. The Department of Radiology handles a substantial volume of emergency imaging cases daily, averaging over 700 CT scans and X-rays per day. As a certified training base for the Ministry of Health-mandated SRT in radiology, the hospital fully meets the qualifications for SRT implementation. It is also designated as the dedicated trauma center, stroke center, and chest pain center of the city, and it provides a wide range of emergency care services.

### Radiologists

Junior radiologists were defined as physicians authorized by the department’s chief radiologist to provide initial interpretations of emergency imaging reports under the senior radiologists’ direct supervision. The group included two trainees who demonstrated outstanding performance and were approved by senior evaluators to participate in emergency shifts; two residents who had completed SRT; and ten attending physicians with varing levels of clinical experience. Among the attending physicians, three had more than 10 years of experience but continued to perform initial reporting duties due to rotational training policies such as rotational requirements or ongoing competency refinement.

Senior radiologists were defined as those approved to independently review reports, including senior attending, associate-chief, and chief radiologists (all with more than 10 years of experience). This authorization was granted based on clinical performance evaluations (e.g., diagnostic accuracy and report comprehensiveness) and operational need rather than strict years of experience.

Due to differences in training approaches, none of these radiologists had undergone subspecialty training. They take turns in interpreting reports across multiple subspecialties and various anatomical regions of images.

### Training and continuing education

Trainees at our institution participated in China’s SRT program, a structured three-year curriculun featuring progressive training and supervision.

#### Year 1

The first year focused on developing foundational knowledge and skills in emergency radiology, general radiography, and cross-sectional imaging (e.g., CT, MRI). Trainees independently completed initial diagnoses for basic imaging cases, with senior radiologists providing step-by-step guidance to improve report quality.

#### Year 2

Trainees engaged in clinical practice under the guidance of senior radiologists, gaining comprehensive exposure to system-specific imaging features and gradually mastering diagnostic strategies for common diseases. This training phase primarily evaluates trainees’ integrated understanding of anatomical regions and high-prevalence pathologies, rather than pursuing specialized subspecialty certification.

#### Year 3

Trainees engaged in supervised independent practice, participating in the diagnosis and discussion of complex and critical cases, while mastering self-learning and research methodologies. By the end of this stage, they were expected to demonstrate basic competence in independent clinical radiology practice and successfully complete standardized competency evaluations.

For radiologists who completed SRT, continuing education relied on self-directed learning, daily morning meetings, case-based discussions, and academic conferences. Supervision intensity was empirically adjusted based on individual performance to ensure diagnostic accuracy.

### Time periods and shift

From January 2023 to March 2024, shifts were randomly selected using computer-generated random numbers (IBM SPSS Statistics 26.0), with stratification by day/night to ensure balanced representation. Each shift was assigned a unique identifier, and shifts were selected without replacement to minimize selection bias related to timing or radiologist assignment. Emergency day shifts ran from 8:00 AM to 4:00 PM and were divided into two periods: early day shift (EDS; 8:00–10:59) and late day shift(LDS; 11:00–15:59). Night shifts ran from 8:00 PM to 8:00 AM and were divided into two segments: early night shift (ENS; 20:00–1:59) and late night shift (LNS; 2:00–7:59). Since dedicated mid-shift radiologists were assigned from 4:00 PM to 8:00 PM, this period was excluded from the analysis in the present study. Junior and senior radiologists have their own rotation schedules, with each group taking turns on night shifts. Due to different numbers of personnel, the senior radiologist paired with a junior radiologist may vary for each shift. All radiologists were required to perform regular outpatient duties, except during scheduled emergency shifts and rest days.

During emergency shifts, junior radiologists are responsible for the initial interpretation of all emergency imaging reports. The workload for each shift is calculated by dividing the total number of reports by the shift duration. Senior radiologists review all emergency imaging reports during their shifts to ensure timely and accurate finalization before clinical release. Any urgent corrections are immediately communicated to the clinical team. All radiologists have at least 24 h of rest before night shifts, while day-shift radiologists participate in regular outpatient duties without special rest arrangements.

To quantify workload intensity, we calculated both total examination counts and hourly workload density for different imaging modalities and body parts. For imaging modality-level analysis, we calculated the hourly examination counts (*count_per_hour*) by dividing the total number of examinations (*count*) in a given period by the product of the period duration in hours (hours_per_period) and the number of shifts (*shift_count*). For example, the hourly CT workload was calculated as:

$$\displaylines{CT\:count\:per\:hour = \frac{{total\:CT\:exams}}{{hours\:per\:period * day\:shifts\:counts}}}$$

Similarly, for body part-level analysis, the hourly workload for chest CT was:

$$\displaylines{Chest\:CT\:count\:per\:hour = \frac{{total\:chest\:CT}}{{hours\:per\:period * day\:shifts\:counts}}}$$

Additionally, to assess inter-shift variability, the number of reports for each shift was recorded, including the total number of reports and hourly workload for each day and night shift, to demonstrate the workload fluctuations across different shifts. This approach enabled us to characterize workload density across shift types (day/night) and periods (early/late), accounting for both the volume of examinations and the temporal distribution of workload. All calculations were performed using aggregate data to protect individual radiologist privacy, with no radiologists linked to workload metrics.

### Differences in interpretation

Discrepancies in interpretation were referred to as the “correction rate” rather than the “error rate” for rigor. To minimize bias, all modifications were manually reviewed by two senior radiologists (each with > 10 years of experience), who excluded any changes attributable solely to stylistic or wording variations from the correction analysis. Discrepancies in interpretation were defined as modifications made by the senior radiologists to the initial report provided by the junior radiologists, where such changes could potentially impact clinical management or lead to controversy. For example, these discrepancies include missed diagnoses of pneumonia or fractures on DR, incorrect localization of intracranial hematomas or missed diagnoses of brain herniation on CT. Reports without further clinically meaningful corrections after retrospective evaluation were established as the final reference standard.

### Statistical analysis

Workload variables were analyzed using descriptive statistics, including mean hourly examination counts for different imaging modalities (e.g., CT and radiography) and anatomical regions. To assess inter-shift variability, we calculated the coefficient of variation (CV, defined as standard deviation/mean×100%) for total reports and hourly workloads across day/night shifts. A generalized estimating equation model assessed revision rate differences between day and night shifts, with shift time as the variable of interest. Chi-square tests evaluated differences in revision rates between the early and late periods of shifts. The relationship between radiologist experience and correction rates was analyzed using the chi-square test and Spearman’s rank correlation coefficient. Patient age and correction rates were analyzed with Kruskal-Wallis tests, followed by pairwise comparisons using the Nemenyi test from the PMCMRplus package.

For the comparison of hourly workload between day and night shifts, we first verified the normality of each group using the Shapiro-Wilk test. If normality was satisfied, Levene’s test was then used to verify the homogeneity of variances. If both assumptions were met, an independent samples t-test was used; otherwise, Welch’s t-test was applied. For non-normal data, the Mann-Whitney U test was used when distribution shapes were similar; otherwise, other appropriate nonparametric tests were selected.

Analyses were performed using IBM SPSS Statistics for Windows, version 26.0 (IBM Corp.) and R version 4.3.3 (R Foundation for Statistical Computing, Vienna, Austria). Statistical significance was defined as *P* < 0.05.

## Results

Among 8338 ER reports initially interpreted by 14 junior radiologists (Table 1) across 19 daytime and 18 nighttime shifts, the correction rate for daytime reports was 25.09%, and for nighttime reports, it was 21.91% (*p* = 0.24, not statistically significant; Table 2). All reviewed and corrected reports had no further post-review modifications. Notably, more radiologists had a lower correction rate at night (10/14, 71.43%) compared to the day (4/14, 28.57%). Among these, the average nighttime interpretation correction rate of radiologists with 3 years of experience (15.12%) was lower than that of those with over 10 years of experience (35.77%).

**Table 1:** Basic information of 14 junior radiologists

Information	Label	Value
Sex	Male	4
	Female	10
Experience	0–4	4
	5–9	7
	10–14	3
SRT Certificate	Yes	12
	No	2
Job Title	Attending	10
	Resident	2
	Trainee	2

Note.—SRT: the Standardised Residency Training

**Table 2:** Day and night shift report correction rates for 14 junior radiologists

Radiologists	Day Correction Rate (%)	Night Correction Rate (%)	Difference (%)
R01	22.39 (105/469)	19.94 (65/326)	-2.45
R02	25.58 (110/430)	15.12 (62/410)	-10.46
R03	23.79 (69/290)	22.14 (60/271)	-1.65
R04	24.79 (60/242)	28.57 (66/231)	3.78
R05	21.05 (48/228)	11.20 (56/500)	-9.85
R06	61.33 (138/225)	50.18 (143/285)	-11.15
R07	28.40 (115/405)	14.57 (80/549)	-13.83
R08	22.22 (48/216)	17.24 (40/232)	-4.98
R09	12.72 (22/173)	3.59 (8/223)	-9.13
R10	20.24 (51/252)	18.15 (51/281)	-2.09
R11	30.43 (63/207)	26.09 (48/184)	-4.34
R12	15.66 (39/249)	25.64 (70/273)	9.98
R13	25.41 (46/181)	35.77 (88/246)	10.36
R14	20.35 (82/403)	33.61 (120/357)	13.26
Overall	25.09 (996/3970)	21.91 (957/4368)	-3.18

Note.—Difference calculated by subtracting the day correction rate from the night correction rate. Data in parentheses are the ratio used to calculate the percentages

The correction rate for LDS (22.15%) was significantly lower than for EDS (28.89%; *p* < 0.001), and the rate for LNS (19.05%) was lower than for ENS (22.89%; *p* = 0.008; Table 3). Overall, 9 of 14 radiologists had lower correction rates for LNS compared to ENS (Table S1).

**Table 3:** Correction rates based on time period of initial interpretation

Time Period	Total	No Correction	Correction	Percentage Correction(%)	*p*
Day assignment					
8:00 AM to 10:59 AM	1731	1231	500	28.9	0.000
11:00 AM to 15:59 PM	2239	1743	496	22.2	
Night assignment					
20:00 PM to 1:59 AM	3255	2510	745	22.9	0.008
2:00 AM to 7:59 AM	1113	901	212	19.0	

Out of 8338 reports, 1953 initial diagnoses were corrected by senior radiologists, with an overall correction rate of 23.42%. Most corrections (76.65%) involved CT scans, and among these, 79.37% were made during CT interpretations (Table 4). The majority of corrections in CT interpretations pertained to the abdomen and pelvis and chest (79.74%), whereas for radiography interpretations, the chest and limbs accounted for 92.77% (Table S2). After standardizing for volume, the lowest correction rates for CT interpretations were observed in the abdomen and pelvis (6.97%) and chest (8.75%). For radiography interpretations, the lowest correction rates were found in the limbs (10.83%) and the chest (27.97%), excluding the neck region (Table S3).

**Table 4:** Breakdown by modality and body parts for all examinations

Discrepancies per Modality	Value
Overall	
No.of examinations	8338
No. of major discrepancies	1953
Discrepancy rate (%)	23.42[1953/8338]
CT	
Body Parts	
Abdomen and pelvis	2618
Chest	2288
Head	1147
Spine	199
Limbs	84
Neck	55
No. of examinations	6391
No. of major discrepancies	1550
Discrepancy rate (%)	24.25[1550/6391]
Total discrepancies (%)	79.37[1550/1953]
Radiography	
Body Parts	
Limbs	1061
Chest	756
Spine	65
Abdomen and pelvis	59
Neck	1
No. of examinations	1942
No. of major discrepancies	401
Discrepancy rate (%)	20.65[401/1942]
Total discrepancies (%)	20.53[401/1953]
MRI	
Body Parts	
Head	4
Chest	3
No. of examinations	7
No. of major discrepancies	2
Discrepancy rate (%)	28.57[2/7]
Total discrepancies (%)	0.10[2/1953]

No significant differences were observed in initial report correction rates based on radiologist professional title, but work experience showed a significant impact (Table S4). The correction rates for radiologists with ≤ 4 years of experience were 22.18% and 22.99%, while the correction rate for radiologists with 13 years of experience was 31.38%.

Night shifts showed a lower volume of reports interpreted compared to day shifts (20.2 vs. 26.1 studies per hour, Table S5), primarily driven by reduced examinations during the second half of night shifts. While ENS required interpreting 30.1 reports per hour, which was slightly lower than EDS (30.3 reports per hour, Table S5), LNS saw a marked decline to 10.3 reports per hour, contrasting with 23.6 reports per hour in the LDS.

**Fig. 1:**
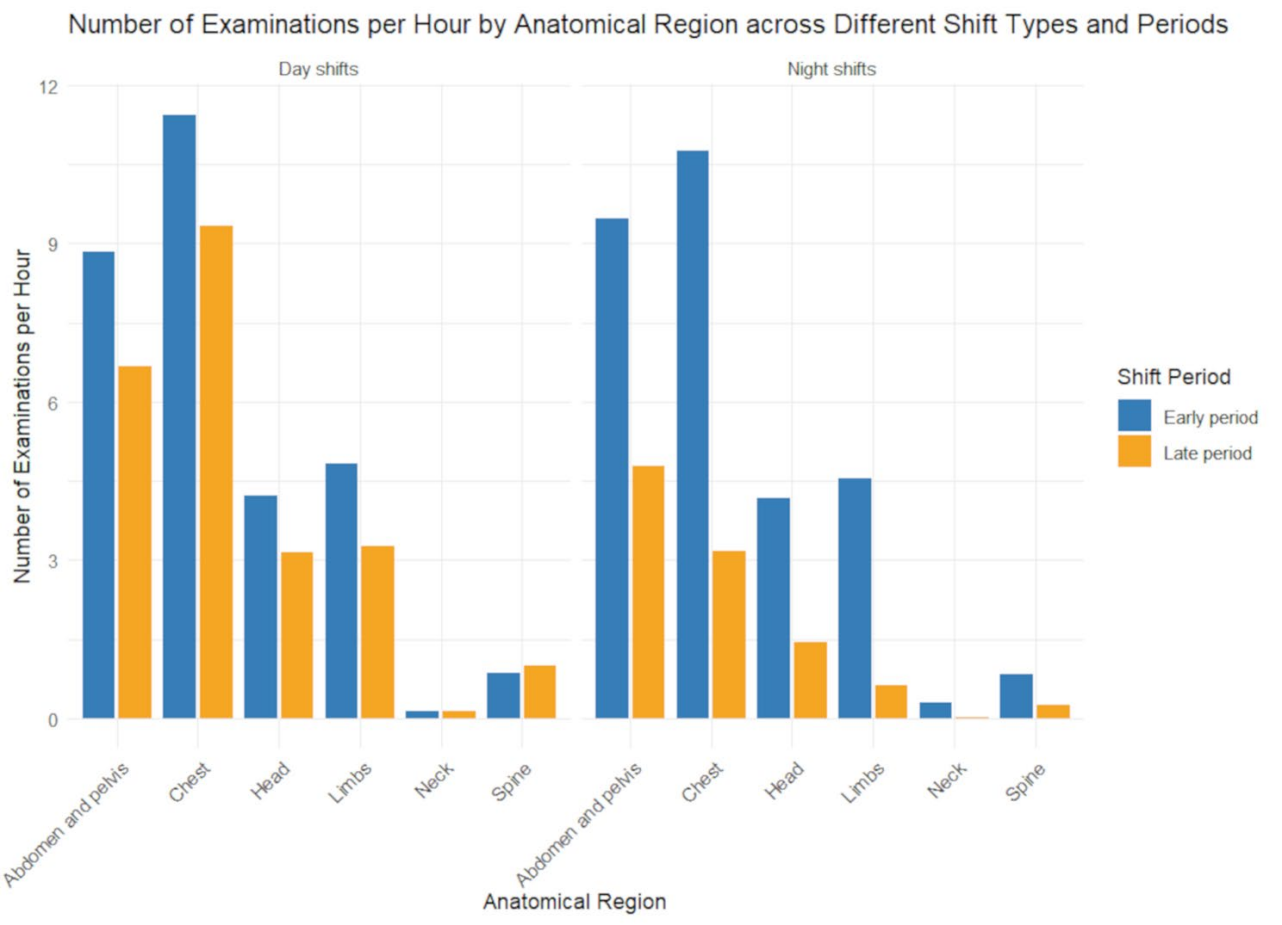
Number of Examinations per Hour by Anatomical Region across Different Shift Types and Periods

**Fig. 2:**
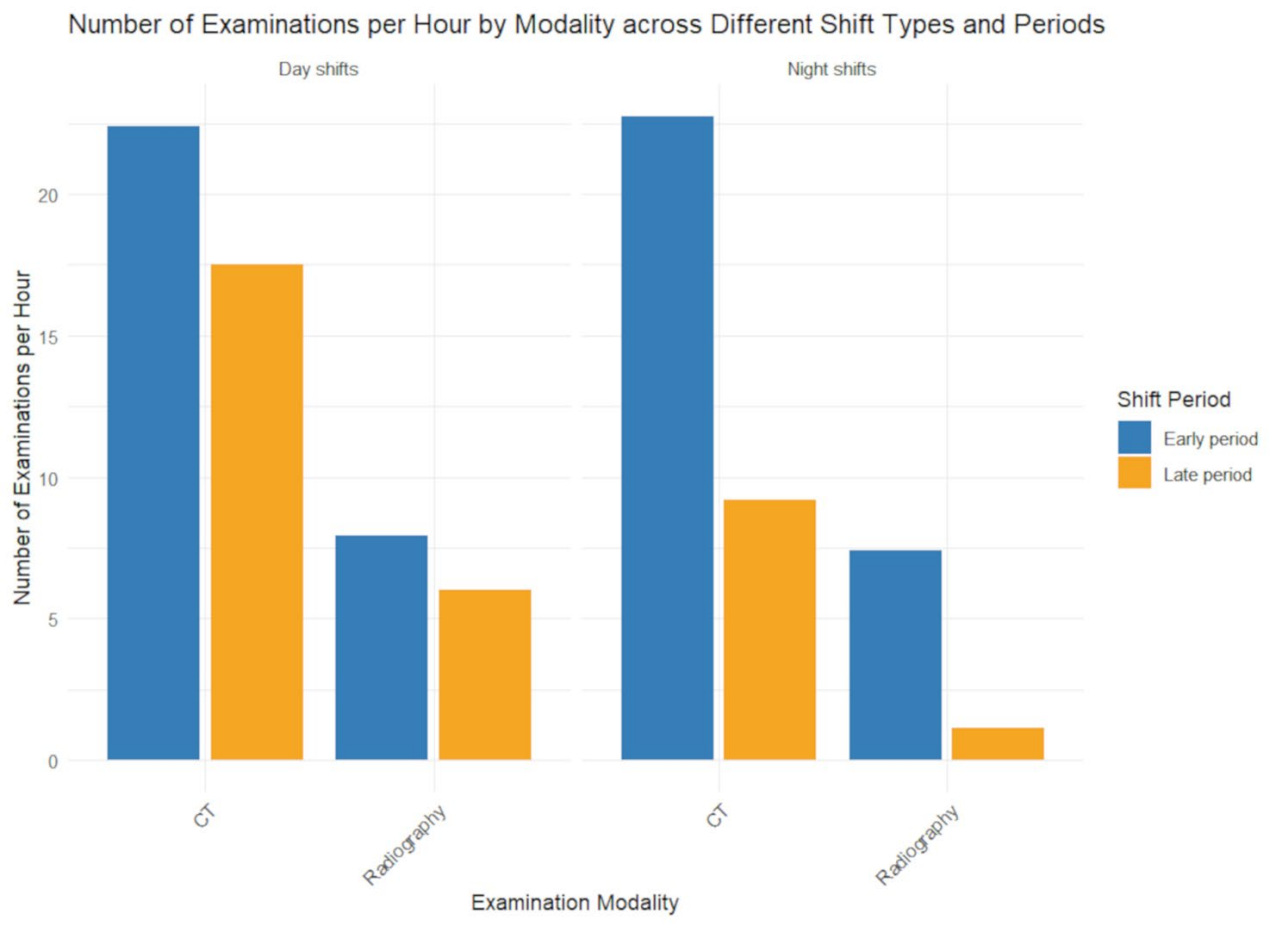
Number of Examinations per Hour by Modality across Different Shift Types and Periods

Notably, ENS featured 10.8 chest studies and 9.5 abdomen and pelvis studies per hour (Table S6), slightly exceeding daytime workloads for these complex regions (11.4 and 8.8 studies per hour, respectively, Fig. 1). Despite overall lower night-shift volumes, late night shifts still demanded interpreting one report every 6 min on average. During this period, CT workload dropped to 9.2 studies per hour (Fig. 2), but abdomen and pelvis (4.8 studies per hour) and chest (3.2 studies per hour) exams remained elevated (Table S6, Fig. 1). This aligns with the observed decline in correction rates from 22.9 to 19.0% (Table 3), as the reduction in total reports, despite persistent high-complexity cases, contributed to the overall correction rate decrease (Table S2).

**Fig. 3:**
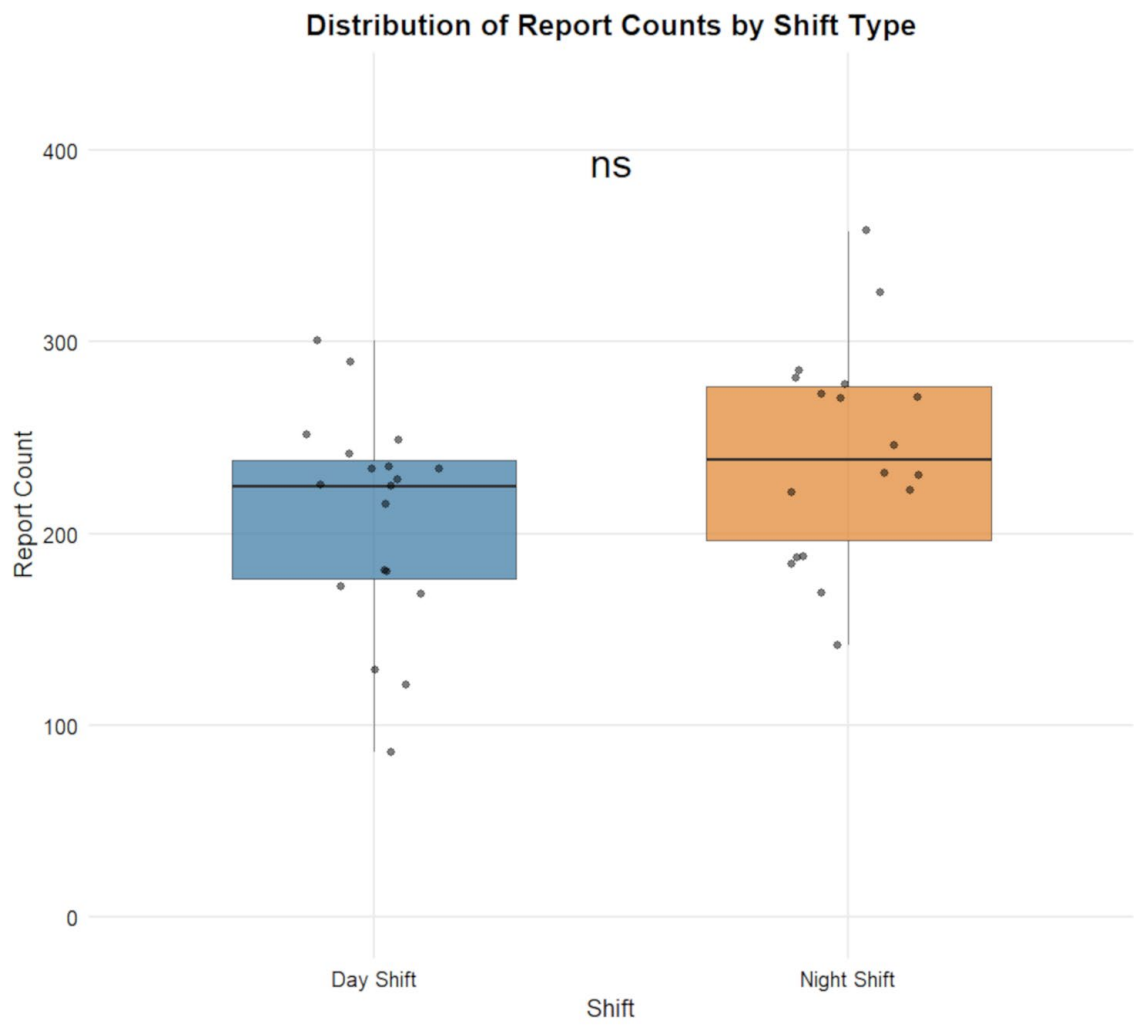
Distribution of Report Counts by Shift Type Note.—Data are presented as mean ± SD; ns indicates no significant difference between groups as determined by two-sample t-test

**Fig. 4:**
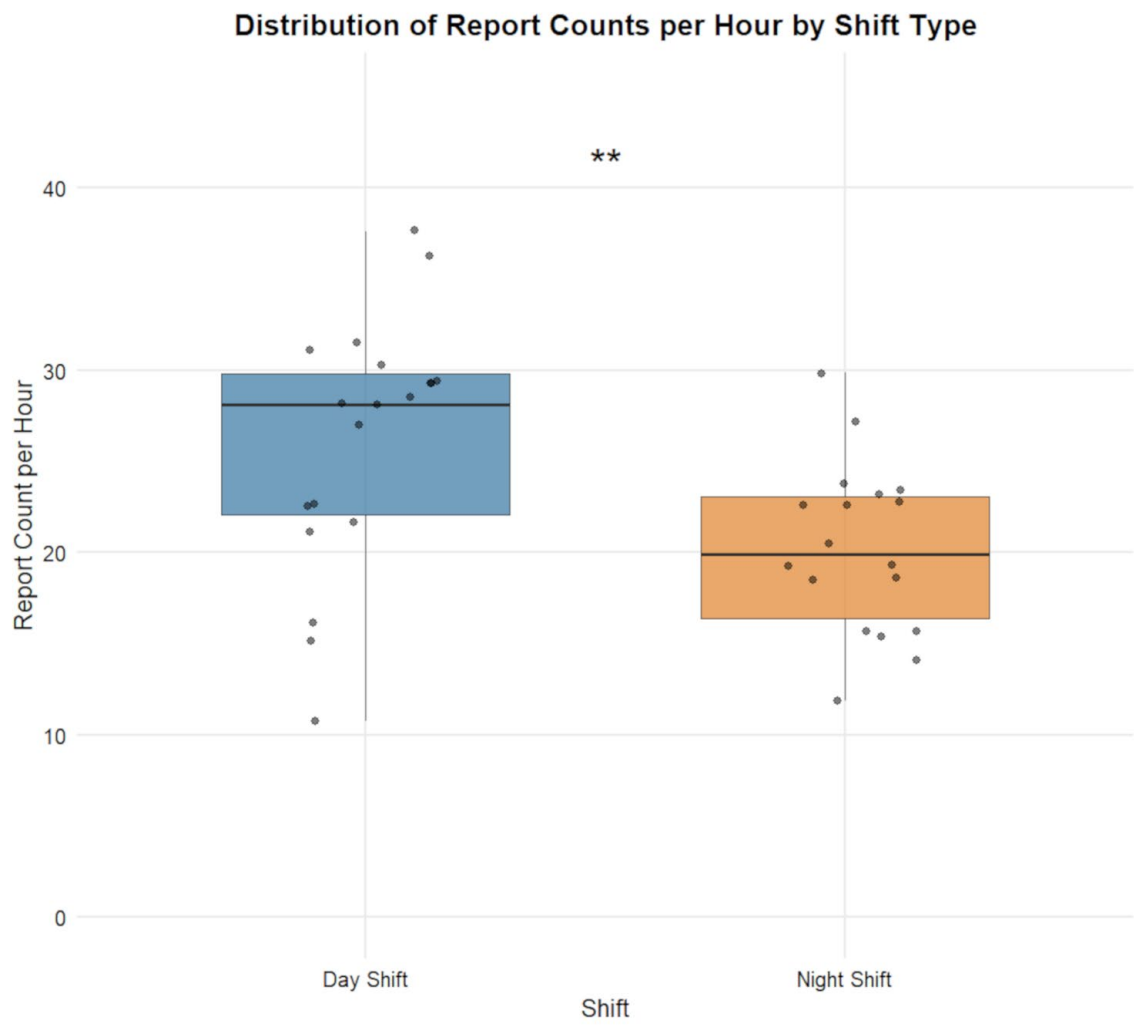
Distribution of Report Counts per Hour by Shift Type Note.—Data are presented as mean ± SD; **: *p* < 0.01

Considering inter-shift variability, we also tabulated the total number of reports and hourly report counts for each shift (Table S7). Table 5 shows that day shifts (*n* = 19) had a mean total report volume of 208.95 (SD = 55.88, CV = 26.74%), while night shifts (*n* = 18) had a mean of 242.67 (SD = 56.01, CV = 23.08%), with no statistically significant difference between the two (*p* = 0.07) (Fig. 3). The mean hourly workload for day shifts was 26.12 reports (SD = 6.98, CV = 26.74%), significantly higher than that for night shifts (20.22 reports, SD = 4.67, CV = 23.08%, t = 3.00, *p* = 0.005) (Fig. 4).

**Table 5:** Descriptive statistics of report volumes and hourly counts for day and night shifts

Shift	Mean	SD	CV (%)	Mean per hour	SD per hour	CV per hour (%)
day shift	208.95	55.88	26.74	26.12	6.98	26.74
night shift	242.67	56.01	23.08	20.22	4.67	23.08

Note.—Report volumes per hour were calculated by dividing the total number of reports by shift duration (8 h for day shift and 12 h for night shift). Data are presented as mean, standard deviation (SD) and coefficient of variation (CV)

Patient age was collected from 5,982 reports, while patient gender data was gathered from 5,924 reports out of the total 8,338. A significant correlation was found between patient age and report correction rates (*p* < 0.001) (Table S8). Elderly patients had significantly higher correction rates than children (*p* = 0.004) and middle-aged patients (*p* < 0.001), while no significant differences were observed between children and middle-aged patients. Gender did not significantly affect correction rates (*p* = 0.06).

## Discussion

**Fig. 5:**
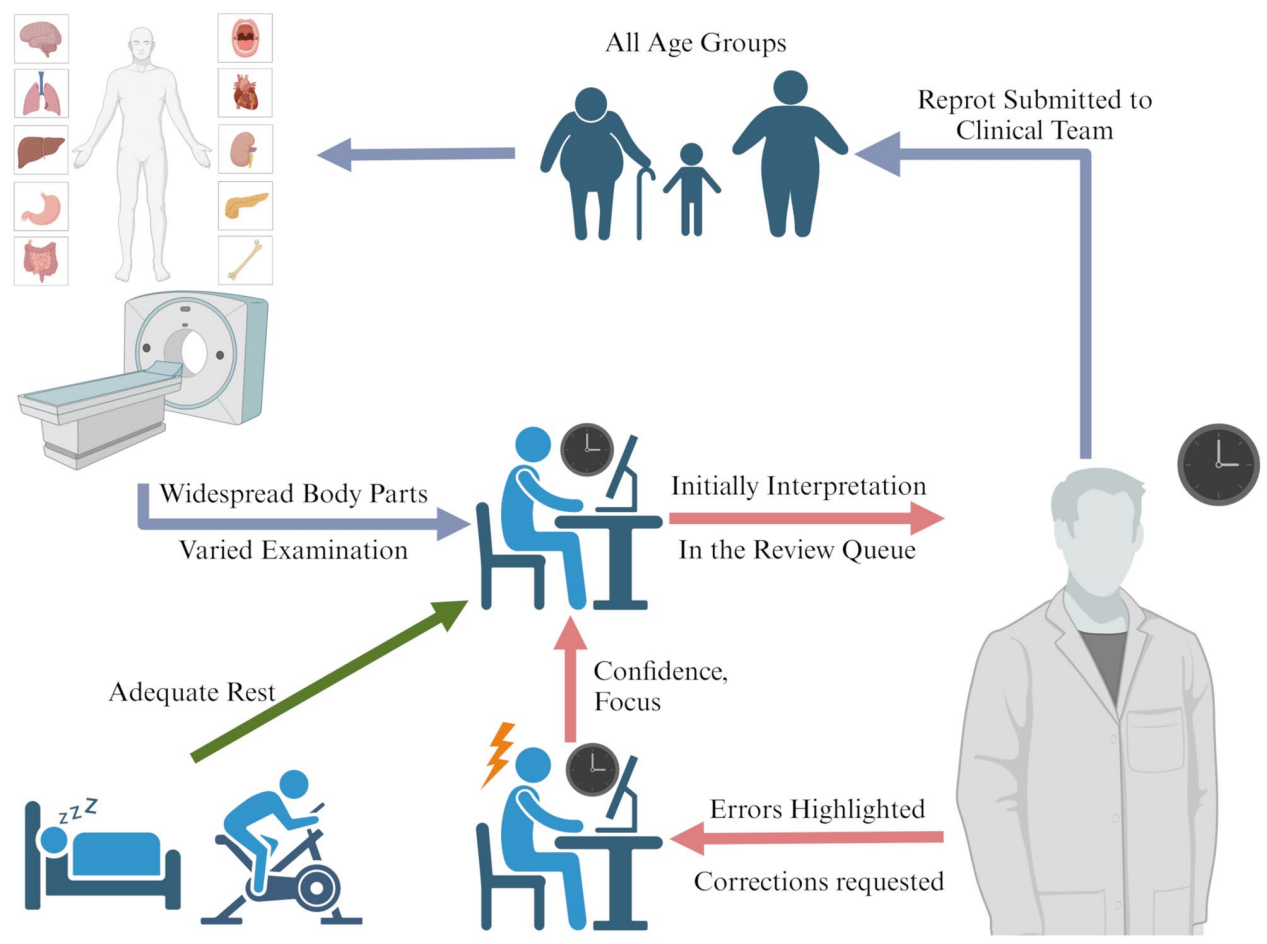
Radiologists’ Overall Workflow During Emergency Duty Shifts Created with BioRender.com

Previous research has consistently shown that error rates increase with longer shift hours. Patel et al. found that errors in interpreting CT scans were more frequent at night, particularly in the later hours of the night shift [2]. Another study quantified radiologists’ fatigue and found that the similarity of resident interpretations decreased as shift duration increased [10]. In contrast, we observed that the correction rate for emergency reports was lower at night than during the day, although the difference was not significant. This discrepancy may relate to two interrelated factors: first, night shifts featured lower hourly workloads (20.22 vs. 26.12 reports/hour, Table 5), enabling more deliberate interpretation despite comparable early shift workloads (30.1 vs. 30.3 reports/hour, Table S5). Second, our scheduling protocols may have mitigated fatigue-related performance decrements. Even though junior radiologists may exhibit higher energy levels initially, prolonged shifts pose risks. Research links circadian disruption to increased diagnostic errors [11, 12]. To address this, our institution mandates no more than 12 consecutive working hours, with 24 h of rest before night shifts and 48 h of downtime post-shift (Fig. 5). The structured rest protocols coincided with stable correction rates in late night shifts (19.05%, Table 3), suggesting scheduled rest offsets fatigue [13].

Our findings indicate that both day and night shifts experienced a notable decline in correction rates during the latter half of the shifts. The night shift showed a lower correction rate initial interpretations compared to the day shift. We propose that one potential explanation for this pattern is the reduced frequency of non-clinical interruptions during night shifts, which may allow radiologists to maintain greater focus during consecutive interpretations [12]. Additionally, the gradual acclimatization of radiologists at the beginning of a shift may contribute to improved performance later on. As radiologists adjust to the work environment, process an increasing number of cases, and receive feedback from senior colleagues, their diagnostic efficiency and accuracy tend to improve over the course of the shift (Fig. 5). However, prior research highlights that the underlying mechanisms are complex and multifactorial. Factors such as shift timing, frequency, and duration are significant independent predictors of radiologists’ performance and may interact in intricate ways [14]. For instance, senior radiologists might exhibit more meticulous review habits during early shift periods, with review rigor potentially decreasing as fatigue accumulates [15]. This aligns with prior research showing that radiologist attention spans decline linearly with shift duration, leading to less thorough error detection [10]. Prior to more targeted research, we cannot rule out the possibility that this pattern may also be related to a decrease in review rigor by senior radiologists over time. These competing hypotheses—feedback mechanism, reviewer fatigue, learner adaptation, and nocturnal interruption differences—warrant disentanglement in future studies.

Notably, early day and night shifts exhibited similar workload densities (30.3 vs. 30.1 reports/hour, Table S5), yet night shifts showed lower correction rates (21.91% vs. 25.09%, Table 2)—a paradox likely explained by reduced interruptions from outpatient consultations or administrative tasks and more stable workloads (lower CV, Table 5). Indeed, despite the longer duration of night shifts, the mean hourly workload was significantly lower than that of day shifts (20.22 vs. 26.12 reports/hour, t = 3.00, *p* = 0.005, Table 5), suggesting a less intense pace that may allow for more deliberate interpretation. This workload parity dissolved in late shifts: while LNS workload dropped to 10.3 reports/hour, abdomen/pelvis (4.8 studies/hour) and chest exams (3.2 studies/hour) remained elevated (Table S6), with the majority being CT scans (9.2 studies/hour) rather than radiography (1.1 studies/hour; Table S5). This observation illustrates a “volume-complexity trade-off”: fewer total cases allowed for longer interpretation times (5.8 vs. 2.5 min per study), potentially offsetting the cognitive demands associated with complex CT scans (Table S3). Nevertheless, despite this compensatory mechanism, CT scans still accounted for the highest proportion of corrections (79.37%, Table 4), primarily due to the inherent complexity of multi-dimensional image analysis. This aligns with prior research showing increased night-shift CT interpretation errors [2], and is further supported by broader trends: in recent years, the demand for CT scans in clinical practice has surged, adding considerable pressure on radiologists [16, 17]. Our data also revealed a more nuanced pattern: while abdominal/pelvic and chest CT scans had the highest absolute correction rates, their standardized rates declined after adjusting for workload (6.97% and 8.75%; Table S3). This “practice makes perfect” effect suggests that high-volume exposure to complex regions can transform challenge into proficiency, though absolute correction rates remain significant due to clinical demand.

To address this issue, implementing tailored training in high-correction areas such as chest CT interpretation could help optimize junior radiologists’ diagnostic skills. Our findings suggest that such interventions may reduce the need for revisions. For example, chest and limb errors accounted for 92.77% of all radiography corrections (Table S2), and addressing these common errors could substantially alleviate the review burden on senior radiologists [18]. Notably, Chinese radiologists face extremely high workloads during night shifts, with an average of 20.22 reports interpreted per hour. Even in the least busy night shifts, the workload still reached 11.83 reports per hour, which is nearly 6.5 times higher than U.S. institution in a study [2]. For context, Frank et al. recommended a maximum shift length of 10 h, with hourly limits for different report types to ensure diagnostic accuracy and prevent fatigue-related errors [19]. Yet, junior radiologists maintained comparable performance despite these demands, highlighting a potential balance between experience and workload-induced fatigue that warrants further investigation.

This study revealed a significant association between radiologists’ clinical experience and emergency report correction rates, though the small sample size precluded calculating proportional relationships. Trainees with 3 years of experience exhibited a correction rate of 22.18%, lower than the 31.38% observed among radiologists with 13 years of experience (Table S4). Notably, we defined “correction rate” as modifications that influence clinical management, specifically excluding stylistic or non-clinically relevant variations in interpretation. This definition ensures that our findings reflect genuine diagnostic discrepancies rather than individual reporting preferences. Despite this rigorous criterion, more experienced junior radiologists still demonstrated higher correction rates. This phenomenon may be attributed to China’s SRT system, which provides junior radiologists with systematic 3-year curricula and intensive supervision to form standardized diagnostic frameworks. By contrast, a few longer-experienced radiologists relying on intermittent learning (e.g., weekly conferences) may struggle to address evolving diagnostic challenges, potentially contributing to higher correction rates. Younger radiologists also demonstrate stronger plasticity, adapting more readily to senior radiologists’ diagnostic habits through training. In contrast, junior radiologists with longer experience may develop individualized interpretive approaches that gradually deviate from clinical consensus. These idiosyncratic frameworks, combined with prolonged exposure to high workloads, may increase susceptibility to fatigue-related errors [6]. Unlike Western systems with subspecialty segmentation [1, 20], China’s non-subspecialized radiology training enriches practical experience but may compromise diagnostic consistency in complex anatomical regions, as reflected by the 79.74% correction rate for abdominal and chest CTs (Table S2). However, the small sample size and aggregate data analysis (to protect privacy) limit granular interpretation of training-accuracy associations. Future multi-center studies tracking standardized training metrics are needed to validate these findings and refine continuing education strategies for different experience levels.

This study found a significant association between patient age and emergency reports correction rates (Table S7). Older patients exhibited a notably higher correction rate (29.85%) compared to children (22.03%) and middle-aged adults (18.85%). This disparity likely stems from age-related diagnostic complexities. Elderly patients often present with extensive medical histories, multiple comorbidities, and atypical disease manifestations, while age-related anatomical changes and degenerative conditions may obscure pathological findings. In contrast, patient gender did not significantly impact the correction rate of ER reports in this study. These findings underscore the unique challenges presented by the elderly patient population, especially for junior radiologists with less practical experience. During emergency shifts, where elderly patients often present with complex conditions, secondary reviews by senior radiologists play a critical role. These reviews not only ensure patient safety but also provide invaluable learning opportunities for junior radiologists, enhancing their vigilance and practical skills through real-time exposure to challenging cases. Tailored training programs addressing specific challenges in imaging elderly patients may be helpful. Additionally, further in-depth research into age-related diagnostic support systems could enhance diagnostic accuracy.

Our study has several limitations. First, the single-center design may limit generalizability to institutions with different scheduling, training systems, or patient demographics. Additionally, as an observational study, these findings require prospective validation to establish causal relationships for workflow recommendations. Second, the retrospective nature of the study introduced potential biases, including selection and information bias. Additionally, the lack of stratification by clinical indication may have introduced unmeasured confounding. For instance, some physicians may have encountered a higher proportion of complex or challenging cases during their shifts, potentially leading to an increased error rate. However, random shift sampling and a large sample size helped mitigate these effects. Third, we did not categorize correction types, limiting insights into specific error impacts. Additionally, adopting senior radiologists’ final interpretations as a reference standard is not infallible. Fatigue, case complexity, and individual cognitive biases may also lead to errors in senior radiologists’ final interpretations. Moreover, while we defined corrections as modifications affecting clinical management, prior research has shown that the proportion of non-clinical stylistic revisions in radiology reports varies widely across studies, ranging from 0.4–22% [18]. This variability highlights that our correction rate might overestimate true diagnostic errors, as subtle style-related discrepancies (e.g., wording or formatting) could have been inadvertently included. The lack of standardized criteria to distinguish ‘clinical errors’ from ‘reporting style’ further limits the precision of our findings. The variability in guidance provided by senior radiologists during their shifts may introduce bias into the correction rates of junior radiologists. Finally, the study cannot provide prescriptive workflow guidelines due to its retrospective nature and single-center scope, which preclude validation of generalizable recommendations. Prospective multi-center trials are needed to translate these findings into evidence-based protocols. Additionally, we did not systematically measure radiologist fatigue, which likely affects interpretation accuracy, especially at night.

In summary, our study demonstrates that, within Chinese hospital settings, junior radiologists’ initial report correction rates were significantly lower during night shifts and later shift periods, despite higher workloads. Radiologist experience and patient age were also found to be significant factors influencing correction rates. These results emphasize the importance of future prospective multicenter studies to rigorously assess how shift scheduling and training frameworks affect diagnostic performance.

## Electronic supplementary material

Below is the link to the electronic supplementary material.

Supplementary Material 1

## Data Availability

The datasets generated were de-identified to protect patient privacy, in accordance with ethical guidelines. Anonymized data are available from the corresponding author on reasonable request, subject to institutional review board approval.

## Abbreviations

ER: Emergency radiology
SRT: Standardised Residency Training
EDS: Early day shift
LDS: Late day shift
ENS: Early night shift
LNS: Late night shift
