# Supplementary material for: Reflections on training and management of junior radiologists through analysis of correction rates in emergency radiology reports
Source: BMC Med Educ. 2025 Jul 1;25:879. doi: 10.1186/s12909-025-07573-1 (PMC12211341; doi:10.1186/s12909-025-07573-1)
Supplement: Supplementary file 1 — Supplementary Material 1 [file 12909_2025_7573_MOESM1_ESM.docx]

**Table S1: Day and Night Half Shift Initial Interpretation Correction Rates for 14 Radiologists**

| Radiologists | EDS Rate (%) | LDS Rate (%) | ENS Rate (%) | LNS Rate (%) |
| --- | --- | --- | --- | --- |
| R01 | 32.74 (55/168) | 16.61 (50/301) | 21.15 (55/260) | 15.15 (10/66) |
| R02 | 28.00 (49/175) | 23.92 (61/255) | 16.40 (52/317) | 10.75 (10/93) |
| R03 | 23.60 (42/178) | 24.11 (27/112) | 23.83 (46/193) | 17.95 (14/78) |
| R04 | 25.00 (28/112) | 24.62 (32/130) | 31.65 (50/158) | 21.92 (16/73) |
| R05 | 17.12 (19/111) | 24.79 (29/117) | 11.17 (40/358) | 11.27 (16/142) |
| R06 | 63.16 (72/114) | 59.46 (66/111) | 48.89 (110/225) | 55.00 (33/60) |
| R07 | 30.18 (83/275) | 24.62 (32/130) | 14.22 (60/422) | 15.75 (20/127) |
| R08 | 21.21 (21/99) | 23.08 (27/117) | 18.24 (31/170) | 14.52 (9/62) |
| R09 | 8.82 (6/68) | 15.24 (16/105) | 4.52 (7/155) | 1.47 (1/68) |
| R10 | 19.74 (15/76) | 20.45 (36/176) | 21.50 (43/200) | 9.88 (8/81) |
| R11 | 39.71 (27/68) | 25.90 (36/139) | 36.51 (46/126) | 3.45 (2/58) |
| R12 | 14.89 (14/94) | 16.13 (25/155) | 28.31 (62/219) | 14.81 (8/54) |
| R13 | 39.19 (29/74) | 15.89 (17/107) | 33.33 (56/168) | 41.03 (32/78) |
| R14 | 33.61 (40/119) | 14.79 (42/284) | 30.63 (87/284) | 45.21 (33/73) |
| Overall | 28.89 (500/1731) | 22.15 (496/2239) | 22.89 (745/3255) | 19.05 (212/1113) |

Note.—EDS: early day shift; LDS: late day shift; ENS: early night shift; LNS: late night shift. Data in parentheses are the ratio used to calculate the percentages.

**Table S2: Breakdown by Modality and Body Parts within the Correction Reports**

| Modality | Body Parts | Rate (%) |
| --- | --- | --- |
| CT | Abdomen and pelvis | 40.71 (631) |
|  | Chest | 39.03 (605) |
|  | Head | 14.06 (218) |
|  | Spine | 3.94 (61) |
|  | Limbs | 1.55 (24) |
|  | Neck | 0.71 (11) |
| Radiography | Chest | 52.62 (211) |
|  | Limbs | 40.15 (161) |
|  | Spine | 4.24 (17) |
|  | Abdomen and pelvis | 2.99 (12) |
| MRI | Head | 1.00 (2) |

Note.—Rates are percentages within their respective modalities, data in parentheses are values for body parts.

**Table S3: Rate and Standardized Rate of Corrections for Different Modalities and Body Parts**

| Modality | Body Parts | Rate (%) | Std Rate (%) |
| --- | --- | --- | --- |
| CT | Abdomen and pelvis | 24.10 (631/2618) | 6.97 |
|  | Chest | 26.44 (605/2288) | 8.75 |
|  | Head | 19.01 (218/1147) | 12.55 |
|  | Spine | 30.65 (61/199) | 116.69 |
|  | Limbs | 28.57 (24/84) | 257.67 |
|  | Neck | 20.00 (11/55) | 275.47 |
| Radiography | Limbs | 15.17 (161/1061) | 10.83 |
|  | Chest | 27.91 (211/756) | 27.97 |
|  | Spine | 26.15 (17/65) | 304.81 |
|  | Abdomen and pelvis | 20.34 (12/59) | 261.15 |
|  | Neck | 0.00 (0/1) | 0.00 |

Note.—Data in parentheses are the ratio used to calculate the percentages. Std Rate: standardized rate.

**Table S4: Interpretation Correction Rates for Different Experience of Junior Radiologists**

| Experience (years) | Correction | Overall | Rate (%)^****^ |
| --- | --- | --- | --- |
| 3 | 407 | 1835 | 22.18 |
| 4 | 129 | 561 | 22.99 |
| 6 | 476 | 1464 | 32.51 |
| 7 | 460 | 2003 | 22.97 |
| 9 | 132 | 929 | 14.21 |
| 13 | 134 | 427 | 31.38 |
| 14 | 215 | 1119 | 19.21 |

Note.—^****^: *p* ＜ .0001.

**Table S5: Total and Average Number of CT and Radiography Studies Interpreted by Time Periods**

| Time Period | CT Count  (per hour) | Radiography Count (per hour) | Combined CT and Radiography Count (per hour) |
| --- | --- | --- | --- |
| Day assignment | 2943 (19.4) | 1024 (6.7) | 3967 (26.1) |
| 8:00 AM to 10:59 AM | 1277 (22.4) | 452 (7.9) | 1729 (30.3) |
| 11:00 AM to 15:59 PM | 1666 (17.5) | 572 (6.0) | 2238 (23.6) |
| Night assignment | 3448 (16.0) | 918 (4.3) | 4366 (20.2) |
| 20:00 PM to 1:59 AM | 2455 (22.7) | 798 (7.4) | 3252 (30.1) |
| 2:00 AM to 7:59 AM | 993 (9.2) | 120 (1.1) | 1113 (10.3) |

**Table S6: Total and Average Imaging Study Interpretation by Anatomical Region and Time Period**

| Time Period | Limbs Count  (per hour) | head Count  (per hour) | chest Count  (per hour) | Spine Count  (per hour) | Abdomen and pelvis Count  (per hour) | Neck Count  (per hour) |
| --- | --- | --- | --- | --- | --- | --- |
| Day assignment | 586 (3.9) | 541 (3.6) | 1538 (10.1) | 145 (1.0) | 1137 (7.5) | 20 (0.1) |
| 8:00 AM to 10:59 AM | 275 (4.8) | 241 (4.2) | 652 (11.4) | 49 (0.9) | 504 (8.8) | 8 (0.1) |
| 11:00 AM to 15:59 PM | 311 (3.3) | 300 (3.2) | 886 (9.3) | 96 (1.0) | 633 (6.7) | 12 (0.1) |
| Night assignment | 559 (2.6) | 566 (2.6) | 1506 (6.97) | 119 (0.6) | 1540 (7.1) | 36 (0.2) |
| 20:00 PM to 1:59 AM | 492 (4.6) | 451 (4.2) | 1163 (10.8) | 91 (0.8) | 1023 (9.5) | 33 (0.3) |
| 2:00 AM to 7:59 AM | 67 (0.6) | 115 (1.4) | 343 (3.2) | 28 (0.3) | 517 (4.8) | 3 (0.0) |

**Table S7: Total Report Count and Hourly Report Count Across All Day and Night Shifts**

| shift | count | count per hour |
| --- | --- | --- |
| Day shift | 235 | 29.38 |
|  | 234 | 29.25 |
|  | 129 | 16.13 |
|  | 301 | 37.63 |
|  | 290 | 36.25 |
|  | 242 | 30.25 |
|  | 228 | 28.50 |
|  | 225 | 28.13 |
|  | 180 | 22.50 |
|  | 225 | 28.13 |
|  | 216 | 27.00 |
|  | 173 | 21.63 |
|  | 252 | 31.50 |
|  | 86 | 10.75 |
|  | 121 | 15.13 |
|  | 249 | 31.13 |
|  | 181 | 22.63 |
|  | 169 | 21.13 |
|  | 234 | 29.25 |
| Night shift | 326 | 27.17 |
|  | 222 | 18.50 |
|  | 188 | 15.67 |
|  | 271 | 22.58 |
|  | 231 | 19.25 |
|  | 358 | 29.83 |
|  | 142 | 11.83 |
|  | 285 | 23.75 |
|  | 278 | 23.17 |
|  | 271 | 22.58 |
|  | 232 | 19.33 |
|  | 223 | 18.58 |
|  | 281 | 23.42 |
|  | 184 | 15.33 |
|  | 273 | 22.75 |
|  | 246 | 20.50 |
|  | 188 | 15.67 |
|  | 169 | 14.08 |

**Table S8: Interpretation Correction Rates Based on Age of Patients**

| Age (years) | Correction | Total | Rate (%)^****^ |
| --- | --- | --- | --- |
| ＜18 | 178 | 808 | 22.03^B^ |
| 18-59 | 661 | 3506 | 18.85^B^ |
| ≥60 | 471 | 1578 | 29.85^A^ |

Note.—A, B: Different letters between groups indicate significant differences (*p* < .01). ^****^: *p* ＜ .0001.
